# Physicochemical Characterization and Antimicrobial Activity against *Erwinia amylovora*, *Erwinia vitivora*, and *Diplodia seriata* of a Light Purple *Hibiscus syriacus* L. Cultivar

**DOI:** 10.3390/plants10091876

**Published:** 2021-09-10

**Authors:** Eva Sánchez-Hernández, Laura Buzón-Durán, Belén Lorenzo-Vidal, Jesús Martín-Gil, Pablo Martín-Ramos

**Affiliations:** 1 Agriculture and Forestry Engineering Department, ETSIIAA, Universidad de Valladolid, Avenida de Madrid 44, 34004 Palencia, Spain; eva.sanchez.hernandez@uva.es (E.S.-H.); laura.buzon@uva.es (L.B.-D.); mgil@iaf.uva.es (J.M.-G.); 2 Servicio de Microbiología, Hospital Universitario Rio Hortega, Calle Dulzaina 2, 47012 Valladolid, Spain; blorenzov@saludcastillayleon.es; 3 Instituto Universitario de Investigación en Ciencias Ambientales de Aragón (IUCA), EPS, Universidad de Zaragoza, Carretera de Cuarte s/n, 22071 Huesca, Spain

**Keywords:** antibacterial, antifungal, bacterial necrosis of grapevine, Botryosphaeria canker, coumarin, fire blight, heptacosanol, Rose of Sharon

## Abstract

Phytochemicals are essential raw materials for the production of formulations that can be helpful in crop protection. In particular, *Hibiscus* spp., which are often used in traditional medicine, are rich in potential bioactive molecules. This study presents an analysis of the thermal, vibrational, and phytochemical characteristics of a light purple variety of *Hibiscus syriacus*, using thermal gravimetric and differential scanning calorimetry, Fourier-transform infrared spectroscopy, and gas chromatography-mass spectroscopy techniques. Further, with a view to its valorization, the antimicrobial activity of its extracts has been investigated in vitro against *Erwinia amylovora* (the phytopathogen responsible for fire blight in apples, pears, and some other members of the family Rosaceae), *Erwinia vitivora* (the causal agent of the “maladie d’Oléron” in grapevines), and *Diplodia seriata* (responsible for “Bot canker”). Higher heating values and thermal features showed similarities with kenaf biomass. The main compounds identified in the hydro-methanolic extracts were: in flowers, 1-heptacosanol, heptacosane, 1-tetracosanol, hexadecenoic acid, 9,12,15-octadecatrienoic acid, and 9,12-octadecadienoic acid; and in leaves, the coumarin derivative 4,4,6,8-tetramethyl-2-chromanone, vitamin E, phytol, and sitosterol. MIC values of 500 and 375 μg·mL^−1^ were obtained against *E. amylovora* for flower and leaf extracts, respectively, upon conjugation with chitosan oligomers (to improve solubility and bioavailability). In the case of *E. vitivora*, MIC values of 250 and 500 μg·mL^−1^, respectively, were registered. Regarding the antifungal activity, EC_90_ values of 975.8 and 603.5 μg·mL^−1^, respectively, were found. These findings suggest that *H. syriacus* (cv. ‘Mathilde’) may be a promising source of antimicrobials for agriculture.

## 1. Introduction

Crop protection is key to global food sustainability and security (in line with Sustainable Development Goal 2 in the 2030 Agenda). Synthetic pesticides have traditionally been used by farmers to control and eradicate pests, but they have detrimental effects on the health of consumers and the environment. To ensure sustainable production patters (SDG Target 12.4) and increase food security, current legislative frameworks promote the use of integrated pest management. In particular, the use of plant extracts as “green agrochemicals” should be intensified. Plants produce a wide range of primary and secondary metabolites (carbohydrates, cyanogenic glycosides, amino acids, lipids, phenols, flavonoids, anthocyanins, alkaloids, and terpenoids, among others) that have bactericidal, fungicidal, virucidal, insecticidal, acaricidal, and nematicidal activities. These phytochemicals are essential raw materials for the production of formulations that can be helpful in crop protection and preservation. However, in spite of the increasing demand for ecofriendly options to manage agricultural pests, the number of botanical-based products remains restricted. The identification of bioactive phytoconstituents in plant extracts thus forms a critical step in the development of commercial biocontrol products, and there is a need for screening promising candidate biorationals.

The genus *Hibiscus* (subkingdom Magnoliophyta, class Magnoliopsida, family Malvaceae), which contains 300 species distributed around the world, constitutes an interesting source of potential bioactive molecules with diverse biological activities, as discussed in the review papers by Vasudeva et al. [1] and Maganha et al. [2]. In fact, a wide range of bioactive phytochemicals have been reported for *H. sabdariffa*, *H. tiliaceus*, *H. rosa-sinensis*, and *H. mutabilis* extracts in the literature [3,4,5,6,7,8,9,10,11,12,13,14].

In the case of *H. syriacus*, the species studied herein, there is less available information. Nonetheless, the presence of nonanedioic acid, suberic acid, 1-octacosanol, *β*-sitosterol, 1,22-docosanediol, betulin, and erythrotriol [15] has been reported for its bark. Methanolic–formic acid extraction of its petals yielded 3-*O*-malonylglucosides of delphinidin, cyanidin, petunidin, pelargonidin, peonidin, and malvidin [16]. A study of its leaves led to the identification of *β*-sitosterol, *β*-daucosterol, *β*-amyrin, oleanolic acid, stigmast-4-en-3-one, friedelin, syriacusin A, kaempferol, isovitexin, vitexin, apigenin, apigenin-7-*O*-*β*-D-glucopyranoside, luteolin-7-*O*-*β*-D-glucopyranoside, vitexin-7-*O*-*β*-D-glucopyranoside, and rutin [17]. More recently, five polyphenols (hydroquinone, naringeninic acid, 4-hydroxybenzaldehyde, vanillic acid, and fumalic acid) and five fatty acids ((2E)-2,6-dimethyl-6-hydroxy-2,7-octadienoic acid, palmitic acid, butyl linoleate, linoleic acid, and stearic acid) were identified in the ethanol extract of the flowers [18]. Hibispeptins A and B [19], triterpene caffeates [20], and syriacusins A-C [21] as antioxidants have been found in the roots, and triterpenoids such as 3*β*-acetoxy-olean-11-en,28,13*β*-olide, 3*β*-acetoxy-11*α*,12*α*-epoxy-olean-28,13*β*-olide, 19*α*-epi-betulin, and 20,28-epoxy-17*β*,19*β*-lupan-3*β*-ol have been identified in the root bark [22].

With regard to the applicability of aforementioned phytoconstituents, studies on the antimicrobial properties of *H. syriacus* extracts have been mostly restricted to human pathogens: for instance, extracts from the whole plant were assessed by Punasiya et al. [23] against *Bacillus cereus*, *Staphylococcus aureus*, and *Klebsiella pneumoniae*, and its seed oil showed activity against *Escherichia coli*, *Salmonella newport*, *S. aureus*, *S. albus*, *B. subtilis,* and *B. anthracis* [24].

Concerning potential applications in crop protection, the fungicidal activity of its seed oil was explored against *Alternaria solani*, *Aspergillus niger*, *Colletotrichum dematium*, and *Fusarium oxysporum* [24]; its bark showed antifungal activity against *Trichophyton interdigitale* [25]; and—in a study of a methanolic extract of roots—activity against *T. mentagrophytes* was reported, which was attributed to nonanoic acid [8]. However, no studies on flower and leaf extracts as biorationals in agriculture have been found after a thorough bibliographical survey.

In view of this research gap, the work presented herein aims to: (i) identify the specific phytochemicals present in the flower and leaf hydromethanolic extracts of *H. syriacus* cv. ‘Mathilde’; and (ii) investigate their antimicrobial activity against apple tree and grapevine pathogens. In particular, against two bacteria—catalogued as quarantine organisms—and a fungus: *Erwinia amylovora* (Burrill) Winslow, Broadhurst, Buchanan, Krumwiede, Rogers and Smith; *Erwinia vitivora* Du Plessis (syn. *Xylophilus ampelinus* (Panagopoulos) Willems, Gillis, Kersters, van den Broeke & De Ley); and *Diplodia seriata* De Not., respectively. Up-to-date information on *E. amylovora*, the causal agent of fire blight—a devastating disease of apples and pears—may be found in the review by Zhao et al. [26]. *E*. *vitivora*, which causes bacterial blight of grapevine (the “maladie d’Oléron” or “mal nero”), results in over 70% harvest losses [27], and its symptoms are often confused with those of “black dead arm” (BDA), caused by Botryosphaeriaceae fungi. Among the latter, *D. seriata* is one of the most abundant, and affects a wide range of woody hosts, including not only grapevine [28,29], but also apples, causing “Bot canker”, frog-eye leaf spot, and black rot [30,31,32].

## 2. Results

### 2.1. Physico-Chemical Characterization

#### 2.1.1. Elemental Analysis and Calorific Values Calculation

The C, H, N, and S percentages of *Hibiscus syriacus* components (wt.% of dry material) were in the 34.4–42.8%, 6.3–6.4%, 2.2–2.8%, and 0.1–0.2% ranges, respectively (Table 1). The distribution of nitrogen content showed maximum values in the flowers and slightly lower in the leaves and stems.

The calculated (from elemental analysis data) higher heating values (HHV) for flowers and leaves were 16.98 and 12.96 kJ·g^−1^, respectively, with a mean value of 14.97 kJ·g^−1^.

#### 2.1.2. Thermal Characterization

The DSC curve for *H. syriacus* flowers (Figure S1) showed exothermal effects at 315, 425, and 443–450–470 °C. The ash content at 550 °C, according to the TG curve, was 6.3%. In turn, the DSC curve of *H. syriacus* leaves showed exothermal effects at 325 and 445 °C. The ash content at 500 °C was 20.6% (Figure S2).

#### 2.1.3. Vibrational Characterization

An inspection of the absorption bands, summarized in Table 2, revealed a composition rich in fatty alcohols, fatty acids, and esters. The broad band at around 3300 cm^−1^ is assigned to the OH stretching vibration, and indicates the presence of primary alcohols. The two intense bands at 2920 and 2850 cm^−1^ are due to CH_2_ asymmetric and symmetric stretching vibrations, respectively. The band at 1734 cm^−1^ is assigned to the C=O stretching vibration of the carboxylic groups in esters. At 1441 cm^−1^, there is a band that can be ascribed to CH_2_ bend (scissors) deformation vibration. Several bands also attributed to CH_2_ vibrations (wagging and twisting) are observed in the 800–1400 cm^−1^ range. The band at 719 cm^−1^, assigned to the CH_2_ rocking mode, is indicative (when it appears together with the other CH_2_ vibrations) of the presence of long-chain linear aliphatic molecules. In the spectrum of leaves, the C=C vibration at 1634 cm^−1^ points to the presence of coumarin derivatives, as discussed below.

#### 2.1.4. Identification of Active Components in the Flower and Leaf Extracts by GC–MS

Among the 43 compounds identified in *H. syriacus* flower hydromethanolic extract (Figure S3, Table 3), the principal constituents were: 1-heptacosanol (*m/z* = 57 and 83) (15.3%) and heptacosane (7%); 1-tetracosanol or lignoceryl alcohol (11%); hexadecanoic acid and its esters (9.6%); 9,12,15-octadecatrienoic acid and its esters (3.5%); 9,12-octadecadienoic acid and its esters (5.2%); 2,3-dihydro-3,5-dihydroxy-6-methyl-4H-pyran-4-one or DDMP-4-one (4%); Z-12-pentacosene (2.5%); and 5-HMF (2.5%). It is worth noting that the methyl esters may be artifacts associated with the use of methanol as the extractive solvent [33].

Concerning the leaf hydromethanolic extracts, in which 27 compounds were identified, the main constituents were: the coumarin derivative 4,4,6,8-tetramethyl-3*H*-chromen-2-one (*m/z* = 162, 189 and 204) (23%); vitamin E homologues (17%); diterpenoid phytol and this acetate (12%); phytosterols as campesterol, stigmasterol, and sitosterol (9%); selinenes (3.5%); squalene (3%); and the methyl esters of 9,12,15-octadecatrienoic acid (2.5%) and 9,12-octadecadienoic acid (2.5%) (Figure S4, Table 4).

#### 2.1.5. Total Polyphenol and Flavonoid Contents

The evaluation of TPC and TFC in the hydromethanolic extracts from *H. syriacus* flowers resulted in 800 mg GAE/100 mg and 315 mg CE/100 mg contents, respectively. As regards the leaf extracts, the TPC and TFC contents were 425 mg GAE/100 mg and 280 mg CE/100 mg, respectively.

### 2.2. Antimicrobial Activity of H. syriacus Extracts and their Phytochemicals

#### 2.2.1. Antibacterial Activity

**Figure 1:**
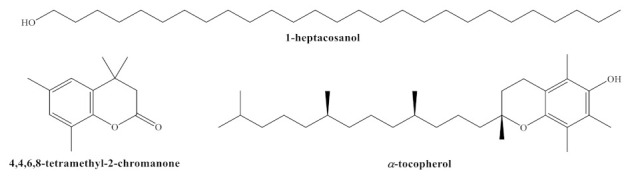
Structures of main phytochemicals found in *H. syriacus* cv. *Mathilde*: 1-heptacosanol; 4,4,6,8-tetramethyl-2-chromanone (or 3,4-dihydro-4,4,6,8-tetramethyl-coumarin, DHTMC); and *α*-tocopherol or vitamin E.

The antibacterial activity against *E. amylovora* and *E. vitivora* of chitosan oligomers (COS), *H. syriacus* flower and leaf hydromethanolic extracts, their main constituents (heptacosanol, DHTMC and vitamin E, Figure 1), and their corresponding conjugate complexes with COS are summarized in Table 5.

Both the flower and leaf extracts showed an antimicrobial activity higher than (or comparable to, in the case of *E. vitivora* for the leaf extract) that of chitosan. Moreover, the flower extract resulted in lower MIC values than those attained with the leaf extract against both pathogens (750 vs. 1000 μg·mL^−1^ against *E. amylovora*, and 500 vs. 1500 μg·mL^−1^ against *E. vitivora*). This is an unexpected result, given that the main constituent of the flower extract (heptacosanol) showed a lower efficacy than the two main compounds present in the leaf extract (DHTMC and vitamin E) in the case of *E. amylovora*, and comparable to that of vitamin E in the case of *E. vitivora*. Hence, other constituents of the flower extract must contribute to its activity, as discussed below.

Upon conjugation with COS, a noticeable enhancement in the antibacterial activity was attained for all the assayed products. In particular, MIC values of 500 and 375 μg·mL^−1^ were obtained against *E. amylovora* for flower and leaf extracts, respectively. In the case of *E. vitivora*, MIC values of 250 and 500 μg·mL^−1^, respectively, were registered. Concerning the main constituents, the best results against *E. amylovora* (MIC = 250 μg·mL^−1^) were registered for COS–vitamin E, while COS–heptacosanol and COS–DHTMC led to the lowest MIC values against *E. vitivora* (187.5 μg·mL^−1^).

#### 2.2.2. Antifungal Activity

**Figure 2:**
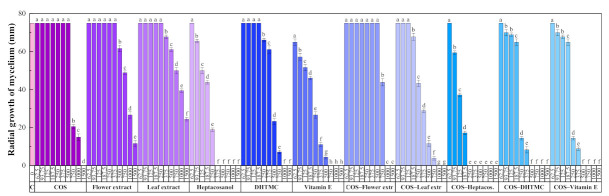
Radial growth of the mycelium for *D. seriata* in in vitro tests conducted in PDA medium with different concentrations (in the 62.5–1500 µg·mL^−1^ range) of chitosan oligomers (COS), *H. syriacus* flower and leaf extracts, their main phytochemical constituents, and their respective conjugate complexes. The same letters above concentrations mean that they are not significantly different at *p* < 0.05. Error bars represent standard deviations.

The results of the antifungal susceptibility test (mycelial growth inhibition using the agar dilution method) are summarized in Figure 2. For all the assayed products, an increase in the concentration led to a decrease in the radial growth of the mycelium, resulting in statistically significant differences.

The two *H. syriacus* extracts showed a lower antifungal activity than COS, for which full inhibition was attained at 1500 μg·mL^−1^. Nonetheless, the main constituents of the extracts, viz. heptacosanol, DHTMC, and vitamin E, showed a stronger antifungal action (reaching full inhibition at 375, 1000, and 750 μg·mL^−1^, respectively).

The formation of conjugate complexes again led to an improvement in terms of antifungal activity: full inhibition was attained at 1000 μg·mL^−1^ for both COS–flower and COS–leaf extracts conjugates (a value lower than that obtained with COS alone), and at 250, 500, and 500 μg·mL^−1^ for COS–heptacosanol, COS–DHTMC, and COS–vitamin E, respectively (Figure S5). This enhancement is clearly observed in the effective concentration values summarized in Table 6.

Calculation of synergy factors, presented in Table 7, confirmed the aforementioned strong synergistic behavior for COS–heptacosanol and COS–vitamin E (with SFs of 2.59 and 3.14 for the EC_90_, respectively). Nonetheless, SFs > 1 were obtained in all cases.

## 3. Discussion

### 3.1. On the Elemental Analysis Results, Calorific Values, and Ash Contents

Regarding the elemental analysis results, upon comparison with those reported for *H. rosa-sinensis* leaves by Subramanian et al. [34] (C, 40.8%; H, 4.7%; N, 4.9%), significant differences in the C/N ratio could be observed (15.6 in this work vs. 8.4 for *H. rosa-sinensis*). In turn, such differences in C and N contents explain the differences in the calorific values: 13 vs. 23.2 kJ·g^−1^. As for the ash content in leaves, the reported content (20.6%) is substantially higher than that found in *H. rosa-sinensis* (12%). Nonetheless, a comparison with *H. cannabinus* (C, 38.3%; H, 5.8%; N; 1.7%) [35] results in a closer match, with a C/N ratio of 22.5, a calorific value of 16.68 kJ·g^−1^ and an ash content of 6.1% (the latter two values are very close to the ones reported for flowers in this work: 16.98 kJ·g^−1^ and 6.3%, respectively). In view of these similarities with *H. cannabinus*, a possible valorization for biofuel production may be explored [36,37].

### 3.2. On the Total Phenol and Flavonoid Contents

The TPC results obtained for *H. syriacus* flower and leaf extracts (800 and 425 mg GAE/100 mg) are within the ranges reported by Wong et al. [38] for the methanolic extracts of other *Hibiscus* species: 264‒2420 mg GAE/100 mg for flowers and 301‒2080 mg GAE/100 mg for leaves, respectively, being close to those found for *H. rosa-sinensis*. The TFC results (315 and 280 mg CE/100 mg for flower and leaf extracts, respectively) were similar to those found after pulsed ultrasonic assisted extraction in methanol of *H. cannabinus* leaves (290 mg CE/100 mg) [39].

### 3.3. On the Composition of H. syriacus Extracts

To date, the only analyses available on *H. syriacus* flower or leaf extracts are those reported by Kim et al. [16] (methanolic formic acid extract of petals, analyzed by ^1^H-NMR and fast atom bombardment mass spectroscopy, FABMS); by Wei et al. [17] (leaf extract, analyzed by ^1^H-NMR and ^13^C-NMR); and by Zhang et al. [18] (ethanolic flower extract, analyzed by ^1^H-NMR and ^13^C-NMR). However, to the best of the authors’ knowledge, no studies based on GC–MS or HPLC are available for *H. syriacus*, so comparisons with other *Hibiscus* spp. extracts are provided instead.

Regarding the main identified flower extract phytoconstituents, 1-heptacosanol has also reported in the essential oil of *H. sabdariffa* flowers by Inikpi et al. [3]. The presence of hexadecanoic and 9,12-octadecadienoic acids and their esters has also been referred in the essential oil of *H. sabdariffa* by Inikpi et al. [3] and in the flowers of *H. tiliaceus* by Melecchi et al. [4]. Concerning the presence of 9,12-octadecadienoic acid (in a 4.4%), it is worth noting that it has also been found by Dingjian et al. [5] in the essential oil of *H. syriacus*. Regarding DDMP-4-one, a principal reducing Maillard compound [40], it has been identified in *H. tiliaceus* [6] and in *H. rosa-sinensis* flowers [7]. In relation to nonanoic acid, albeit present in small amounts (0.82%), it had been previously found in the root of *H. syriacus* [8].

With reference to the phytochemicals found in the leaf extract, coumarin derivatives have been reported in *H. rosa-sinensis* leaf ethanol and water extracts [9]. *α*-tocopherol (vitamin E) has been identified in the ethanolic leaf extract of *H. sabdariffa* by Subhaswaraj et al. [10]. Phytol has been reported in the essential oil of kenaf (*H. cannabinus*) by Kobaisy et al. [11], in the ethanolic leaf extract of *H. sabdariffa* [10], and in the aqueous methanol fraction of *H. asper* leaves by Olivia et al. [12]. In the latter two works, 9,12,15-octadecatrienoic, 9,12-octadecadienoic, and hexadecanoic acids were also found (as in the GC–MS analyses reported herein). *β*-sitosterol has been identified in *H. sabdariffa* and *H. mutabilis* leaves [13,14].

### 3.4. On the Antimicrobial Activity of H. syriacus Extracts

The antibacterial activity of *H. syriacus* extracts has been studied by Punasiya et al. [23] against *B. cereus*, *S. aureus*, and *K. pneumonia*; by Mak et al. [41] against *S. typhimurium* and *S. aureus*; and by Seyyednejad et al. [42] against *B. anthracis*, *B. cereus*, *S. aureus*, *S. epidermidis*, *L. monocytogenes*, *S. pyogenes*, *E. coli*, *S. typhy*, *K. pneumonia*, and *P. aeruginosa*, but no assays against *E. amylovora* and *E. vitivora* pathogens have been carried out. Regarding the antifungal activity, it has been assayed against *C. albicans* and *S. cerevisiae* by Liu et al. [43], and against *T. mentagrophytes* [8], but no data on *Diplodia* spp. (or other *Botryosphaeriaceae*) is available. Hence, a tentative explanation for the observed activity on the basis of the phytoconstituents identified in the extracts is presented.

With respect to the flower extract, 1-heptacosanol has been reported to have antimicrobial and antioxidant activity [44], putative antibacterial activity [45], and significant antifungal activity against all *Candida* spp. [46]. Nonetheless, its efficacy against the phytopathogens referred herein was variable: moderate against *E. amylovora*, and high against *E. vitivora* and *D. seriata*. As regards other constituents that were not assayed in vitro, hexadecanoic acid and its esters are considered antifungals and antioxidants [47]. The same applies to 1-tetracosanol [48,49], and to the unsaturated linolenic and linoleic fatty acids [50,51]. Moreover, according to Čechovská et al. [40], part of the antioxidant activity of *H. syriacus* flowers can be ascribed to 2,3-dihydro-3,5-dihydroxy-6-methyl-4H-pyran-4-one or DDMP-4-one, and such antioxidant activity is generally associated with antibacterial, antifungal, and antimycotoxigenic biological activities [52].

As for the leaf extract, the neoflavonoid 4,4,6,8-tetramethyl-2-chromanone (or 3,4-dihydro-4,4,6,8-tetramethyl-coumarin), although not included among the coumarins screened by Souza et al. [53] against *B. cereus*, *E. coli*, *P. aeruginosa*, and *S. aureus*, nor among those tested by Montagner et al. [54] against *C. albicans*, *A. fumigatus*, and *F. solani*, has shown comparable MIC values to those reported in those works. The lowest efficacy (MIC = 1000 μg·mL^−1^), observed against *E. amylovora*, may still be regarded as moderate, and would support the observations of Halbwirth et al. [55] on the antimicrobial activity of flavonoids in pome fruit trees for fire blight control (contrary to the opinion of Flachowsky et al. [56], who considered that the accumulation of flavanones did not appear to reduce fire blight susceptibility in apple). As a potential explanation behind such activity, the efficacy of phytoalexins and flavonoids may be connected to their capability to elude the outer membrane protein TolC and the AcrAB transport system in *E. amylovora* [57,58].

Regarding other leaf extract constituents, vitamin E is also known to have antimicrobial activity [59,60]. In the present study, vitamin E showed a higher efficacy against *E. vitivora* (MIC = 500 μg·mL^−1^) than against *E. amylovora* and *D. seriata* (MIC = 750 μg·mL^−1^). It should also be taken into consideration that the third main compound, phytol, although not assayed in vitro, may also contribute to the observed antimicrobial activity [61].

In relation to the improved antimicrobial activity of the constituents of *H. syriacus* extracts observed upon conjugation with COS, it may be ascribed to solubility and bioavailability enhancement, as result of an enhanced linkage to negatively charged site-specific binding receptors on the bacterial/fungal membranes. Nevertheless, further research is needed on this specific point, given that no convincing mechanism to explain the synergistic action of above (and other previously reported [62,63]) COS-phytochemical conjugates has been reported to date.

### 3.5. Limitations of the Study

With regard to the evolution of this work, it should be taken into consideration that—even though the in vitro results are promising—in vivo tests are required in order to evaluate the actual field applicability. While no restrictions apply to ex situ and in vivo tests involving *Botryosphaeriaceae* fungi (which may be conducted on autoclaved grapevine wood or on grafted grapevine plants artificially inoculated with the fungal pathogen), bioassays with highly virulent *Erwinia* spp. (for which the best MIC values have been attained and which would be most interesting, given that effective and sustainable control measures are lacking) can only be conducted on suitable host materials under carefully controlled laboratory conditions, given that field studies require authorization, especially in protected zones (according to EU Commission Directive 2003/116/EC of 4 December 2003). Further, even if assays were conducted on artificially inoculated seedlings, it is known that there are sensitivity differences depending on whether it is a natural infection or an artificial inoculation, and also depending on the affected organ (flowers, shoots, unripe fruits, etc.). In addition, comparisons with currently allowed chemical and biological treatment products (viz. Fosetyl-aluminium, laminarin, prohexadione calcium and copper-derivatives; and *Aureobasidium pullulans* and *B. subtilis*) would be needed for the cost-effectiveness analysis.

## 4. Material and Methods

### 4.1. Reagents

Chitosan (CAS 9012-76-4; high MW: 310,000–375,000 Da) was purchased from Hangzhou Simit Chem. & Tech. Co. (Hangzhou, China). Neutrase^TM^ 0.8 L enzyme was supplied by Novozymes A/S (Bagsværd, Denmark). Chitosan oligomers (COS) with a molecular weight of < 2000 Da were prepared according to the procedure reported by Santos-Moriano et al. [64], with the modifications indicated in [65].

1-heptacosanol (CAS 2004-39-9, 98%), 4,4,6,8-tetramethyl-2-chromanone (Aldrich^CPR^ T313513), vitamin E (*α*-tocopherol, CAS 10191-41-0, analytical standard), 2,2-diphenyl-1-picrylhydrazyl (DPPH, CAS 1898-66-4), 6-hydroxy-2,5,7,8-tetramethylchroman-2-carboxylic acid (Trolox, CAS 53188-07-1), methanol (CAS 67-56-1, UHPLC, suitable for MS), TSA (tryptic soy agar, CAS 91079-40-2) and TSB (tryptic soy broth, CAS 8013-01-2) were acquired from Sigma-Aldrich Química (Madrid, Spain). PDA (potato dextrose agar) was supplied by Becton Dickinson (Bergen County, NJ, USA). All reagents were used as supplied, without further purification.

### 4.2. Studied Species

**Figure 3:**
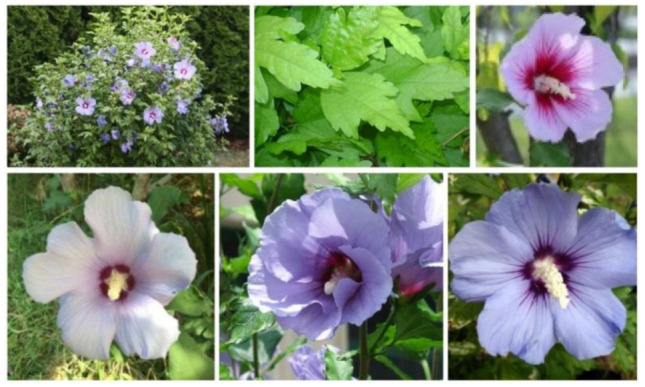
(**Top**) Leaves and flowers of *Hibiscus syriacus*; (**Bottom**) three light purple/purplish white *H. syriacus* cultivars: ‘Mathilde’, ‘Marina’, and ‘Oiseau Blue’ (from left to right).

*Hibiscus syriacus*, colloquially known as ‘Rose of Sharon’ (or ‘Korean Rose’), is one of the 300 species of the genus *Hibiscus*. Although it was first identified in Syria (as indicated by its name), it is mainly found in south-central and southeast China, India, and much of east Asia. This deciduous shrub grows up to 3 m tall and has flowers with attractive white, pink, purple, lavender, or blue color over a long blooming period, though individual flowers last only a day. The leaves are glabrous, triangular-ovate to rhombic, often 3-lobed (Figure 3, top-center).

About 40 different *H. syriacus* cultivars, with varying flower color and shape, are commonly cultivated, and many more genotypes exist in different collections [66]. Among the light purple/purplish white cultivars, ‘Mathilde’ (or Blush Satin^®^), from nursery M. Verweij & Zonen (Boskoop, The Netherlands), released in 1995, is one of the most popular, together with ‘Marina’ cultivar (or Blue Satin^®^), which looks similar to ‘Oiseau Bleu’, but is said to have a stronger growth (Figure 3, bottom). The purple color has been referred to anthocyanin pigments [67].

A pharmacognostic and pharmacological overview of *H. syriacus* is provided in the review paper by Punasiya et al. [68].

### 4.3. Plant Material and Extraction Procedure

*Hibiscus syriacus* cv. ‘Mathilde’ samples (PP12660, Satin^®^ series) were collected in the full flowering stage, in September 2020, in Llanes (Asturias, Spain). A voucher specimen, identified and authenticated by Prof. J. Ascaso, has been deposited at the herbarium of the Escuela Politécnica Superior de Huesca, Universidad de Zaragoza. Aerial parts from different specimens (*n* = 20) were thoroughly mixed to obtain (separate) flowers and leaves composite samples. The composite samples were shade-dried, pulverized to fine powder in a mechanical grinder, homogenized, and sieved (1 mm mesh).

The flower samples were mixed (1:20 *w/v*) with a methanol/water solution (1:1 *v/v*) and heated in a water bath at 50 °C for 30 min, followed by sonication for 5 min in pulse mode with a 1 min stop for each 2.5 min, using a probe-type ultrasonicator (model UIP1000 hdT; 1000 W, 20 kHz; Hielscher Ultrasonics, Teltow, Germany). The solution was then centrifuged at 9000 rpm for 15 min and the supernatant was filtered through Whatman No. 1 paper. Aliquots were lyophilized for CHNS and FTIR analyses. The extraction procedure for leaf samples was identical.

### 4.4. Bacterial and Fungal Isolates

The *E. amylovora* and *E. vitivora* bacterial isolates were supplied by CECT (Valencia, Spain), with NCPPB 595 and CCUG 21,976 strain designations, respectively. The former was isolated from pear (*Pyrus communis* L.) in the UK, and the latter from *Vitis vinifera* var. ‘Sultana’ in Greece. *D. seriata* (code ITACYL_F098, isolate Y-084-01-01a) was isolated from ‘Tempranillo’ diseased grapevine plants from protected designation of origin (PDO) Toro (Spain) and supplied as lyophilized vials (later reconstituted and refreshed as PDA subcultures) by ITACYL (Valladolid, Spain) [69].

### 4.5. Physicochemical Characterization

Elemental analyses of dry ground samples were performed with a LECO (St. Joseph, MI, USA) CHNS-932 apparatus (model No. 601-800-500).

The calculation of calorific values from elemental analysis data was carried out according to the following equation [70]: HHV = (0.341 × %C) + (1.322 × %H) − 0.12(%O + %N), where HHV is the heating value for the dry material, expressed in kJ·g^−1^; and %C, %H, %O, and %N are the mass fractions, expressed in wt.% of dry material.

Thermal gravimetric (TGA) and differential scanning calorimetry (DSC) analyses were conducted with a simultaneous TG-DSC2 apparatus (Mettler Toledo; Columbus, OH, USA). Samples were heated from 30 to 600 °C under N_2_:O_2_ (4:1) flow (20 cm^3^·min^−1^), at a heating rate of 20 °C·min^−1^.

The infrared vibrational spectra were collected using a Thermo Scientific (Waltham, MA, USA) Nicolet iS50 Fourier-transform infrared spectrometer, equipped with an in-built diamond attenuated total reflection (ATR) system. A spectral resolution of 1 cm^−1^ over the 400–4000 cm^−1^ range was used, taking the interferograms that resulted from co-adding 64 scans.

The hydromethanolic plant extracts were studied by gas chromatography-mass spectrometry (GC-MS) at the Research Support Services (STI) at Universidad de Alicante (Alicante, Spain), using a gas chromatograph model 7890A coupled to a quadrupole mass spectrometer model 5975C (both from Agilent Technologies, Santa Clara, CA, USA). The chromatographic conditions were: 3 injections/vial, injection volume = 1 µL; injector temperature = 280 °C, in splitless mode; and initial oven temperature = 60 °C, 2 min, followed by ramp of 10 °C/min up to a final temperature of 300 °C, 15 min. The chromatographic column used for the separation of the compounds was an Agilent Technologies HP-5MS UI of 30 m length, 0.250 mm diameter, and 0.25 µm film. The mass spectrometer conditions were: temperature of the electron impact source of the mass spectrometer = 230 °C and of the quadrupole = 150 °C; and ionization energy = 70 eV. Test mixture 2 for apolar capillary columns according to Grob (Supelco 86501, Sigma Aldrich Química, Madrid, Spain) and PFTBA tuning standards were used for equipment calibration. NIST11 library was used for compound identification.

Total phenolic content, expressed in gallic acid equivalents (GAE), was determined by using the Folin–Ciocalteau method as described by Dudonné et al. [71], and the total flavonoid content, expressed in catechin equivalents (CE), was evaluated according to Mak et al. [42] through the use of the aluminum chloride method. An Agilent UV-Vis Cary 100 spectrometer was used for the colorimetric quantification.

### 4.6. In Vitro Antibacterial Activity Assessment

The antibacterial activity was assessed according to CLSI standard M07-11 [72], using the agar dilution method to determine the minimum inhibitory concentration (MIC). An isolated colony of *E. amylovora* in TSB liquid medium was incubated at 30 °C for 18 h. Serial dilutions were then conducted, starting from a 10^8^ CFU·mL^−1^ concentration, to obtain a final inoculum of ~10^4^ CFU·mL^−1^. Bacterial suspensions were then delivered to the surface of TSA plates, to which the bioactive products had previously been added at concentrations in the 62.5–1500 μg·mL^−1^ range. Plates were incubated at 30 °C for 24 h. In the case of *E. vitivora*, the same procedure was followed, albeit at 26 °C. Readings were taken after 24 h. MICs were visually determined in the agar dilutions as the lowest concentrations of the bioactive products at which no bacterial growth was visible. All experiments were run in triplicate, with each replicate consisting of 3 plates per treatment/concentration.

### 4.7. In Vitro Antifungal Activity Assessment

The antifungal activity of the different treatments was determined using the agar dilution method according to EUCAST standard antifungal susceptibility testing procedures [73], by incorporating aliquots of stock solutions onto the PDA medium to obtain concentrations ranging from 62.5 to 1500 μg·mL^−1^ range. Mycelial plugs (⌀ = 5 mm), from the margin of 1-week-old PDA cultures of *D. seriata*, were transferred to plates incorporating the above-mentioned concentrations for each treatment (3 plates per treatment/concentration, with 2 replicates). Plates were incubated at 25 °C in the dark for a week. PDA medium without any amendment was used as the control. Mycelial growth inhibition was estimated according to the formula: ((*d_c_* − *d_t_*)/*d_c_)* × 100, where *d*_c_ and *d_t_* represent the average diameters of the fungal colony of the control and of the treated fungal colony, respectively. Effective concentrations (EC_50_ and EC_90_) were estimated using PROBIT analysis in IBM SPSS Statistics v.25 (IBM; Armonk, NY, USA) software.

The level of interaction was determined according to Wadley’s method [74], which is based on the assumption that one component of the mixture can substitute at a constant proportion for the other. The expected effectiveness of the mixture is then directly predictable from the effectiveness of the constituents if the relative proportions are known (as it is in this case). The synergy factor (SF) is estimated as:

(1) $$\mathrm{SF}=\frac{ED\left(exp\right)}{ED\left(obs\right)}=\frac{\frac{a+b}{\left(\frac{a}{ED_{A}}+\frac{b}{ED_{B}}\right)}}{ED\left(obs\right)}$$

where *a* and *b* are the proportions of the products A and B in the mixture, respectively, and *a* + *b* = 1; *ED_A_* and *ED_B_* are their equally effective doses; *ED*(*exp*) is the expected equally effective dose; and *ED*(*obs*) is the equally effective dose observed in the experiment. If SF = 1, the hypothesis of similar joint action (i.e., additivity) can be accepted; if SF > 1, there is synergistic action; and if SF < 1, there is antagonistic action between the two fungicide products.

### 4.8. Statistical Analysis

Given that the homogeneity and homoscedasticity requirements were satisfied (according to Shapiro–Wilk and Levene tests, respectively), the mycelial growth inhibition results for *D. seriata* were statistically analyzed in IBM SPSS Statistics (IBM, New York, NY, USA) v.25 software using one-way analysis of variance (ANOVA), followed by post hoc comparison of means through Tukey’s test at *p* < 0.05.

## 5. Conclusions

Elemental and thermal analysis data of *H. syriacus* biomass showed similarities with kenaf, a suitable lignocellulosic feedstock for bioenergy production. The GC–MS analysis of *H. syriacus* extracts revealed that, apart from fatty alcohols and fatty acids, 4,4,6,8-tetramethyl-3H-chromen-2-one, vitamin E (and its precursor phytol), phytosterols, selinenes, 2,3-dihydro-3,5-dihydroxy-6-methyl-4H-pyran-4-one, Z-12-pentacosene, and 5-HMF were also present. The antimicrobial activity of *H. syriacus* extracts was then assayed in vitro. Upon conjugation with COS, flower and leaf extracts led to MIC values of 500 and 375 μg·mL^−1^, respectively, against *E. amylovora*; to MIC values of 250 and 500 μg·mL^−1^, respectively, against *E. vitivora*; and to EC_90_ values of 976 and 604 μg·mL^−1^, respectively, against *D. seriata*. The strong synergistic behavior observed upon conjugation with COS may be ascribed to solubility and bioavailability enhancement. In view of the observed activity, an alternative valorization approach as a source of bioactive products may be envisaged, although in vivo assays are required to determine the actual operational efficacy.

## Figures and Tables

**Table 1:** Elemental (CHNS) composition (wt.%) of flowers and leaves of *Hibiscus syriacus*.

Part of the Plant	C	H	N	S	C/N Ratio
Flowers	42.78%	6.4%	2.78%	0.21%	15.4
Leaves	34.38%	6.3%	2.21%	0.07%	15.6

**Table 2:** Main bands in the ATR-FTIR spectra of *Hibiscus syriacus* flowers and leaves and their assignments. Peak positions are expressed in cm^−1^.

Part of the Plant		Assignment
Flower	Leaves	
3289	3335	Bonded O-H stretching (cellulose)
2919	2917	–CH_2_ asymmetric stretching of alkyls (cutine, wax, pectin, fatty acids, and fatty alcohols)
2850	2849	–CH_2_ symmetric stretching (cutine and wax, fatty acids, and fatty alcohols) CH_2_–(C6)—bending (cellulose)
1734	1734	C=O stretching of alkyl ester; and C=O lactone
	1634	C=C in coumarin derivatives; amide I/C=O stretch (hemicellulose, bonded ketones, quinones…).
1607		Aromatic C–C and C=C skeletal stretching; COO—antisymmetric stretching (polygalacturonic and pectin ester); and C=N
1544		Amide II/Aromatic skeletal. Typical of carotenoids.
1441	1443 1417	C=C stretching, aromatics H_2_O vapor; O–CH_3_ stretching; and C–H bending of CH_2_ or CH_3_
1373 1317	1369 1316	
		C–H (cellulose)
1242	1240	Amide III/C–C–O asymmetric stretching acetylated glucomannan; C–O stretching of aryl ether; and C–O and OH of COOH groups
1147	1147	C–O–C in bridge asymmetric (cellulose); C–C in plane (*β*-carotene)
1100	1103	C–O–C stretching in the pyranose ring skeletal (cellulose)
1033	1050	C–H bending vibrations in of planes.
	1019	C–H bending (typical of carotenes); polygalacturonic acid (a variety of pectin in plant cuticles), and pectins. Typical of cyclopropenoid grouping
719	720	CH_2_ rocking

**Table 3:** Main compounds identified in *Hibiscus syriacus* flower hydromethanolic extract by GC–MS.

Peak	R_t_ (min)	Area (%)	Assignments
3	5.085	1.56	2-cyclopenten-1-one, 2-hydroxy
6	6.156	1.95	2-pentanone, 4-hydroxy
7	6.954	1.31	propanal, 2-methyl-, dimethylhydrazone
8	7.563	1.32	3H-pyrazol-3-one, 2,4-dihydro-2,4,5-trimethyl-
9	7.753	1.24	pentanal
11	8.561	4.15	2,3-dihydro-3,5-dihydroxy-6-methyl-4H-pyran-4-one (or DDMP-4-one)
12	8.887	0.65	5,6-epoxy-6-methyl-2-heptanone
14	9.778	2.57	5-hydroxymethylfurfural
15	10.319	0.82	nonanoic acid
17	12.836	1.00	methylparaben
19	18.026	2.59	hexadecanoic acid, methyl ester
20	18.415	5.32	*n*-hexadecanoic acid
21	19.666	3.61	9,12-octadecadienoic acid, methyl ester
22	19.730	2.41	9,12,15-octadecatrienoic acid, methyl ester
23	20.036	1.04	9,12-octadecadienoic acid
24	20.105	1.04	9,12,15-octadecatrienoic acid
25	21.814	0.54	9,12-octadecadienoic acid, methyl ester
28	23.055	1.69	tetracosane
29	23.177	1.67	hexadecanoic acid, 2-hydroxy-1-(hydroxymethyl)ethyl ester
30	24.399	1.46	1-tetracosanol
32	24.564	7.08	heptacosane
35	25.538	0.61	squalene
36	25.830	4.76	1-tetracosanol
37	25.884	4.92	1-tetracosanol
38	25.966	15.27	1-heptacosanol
40	26.653	2.04	*Z*-12-pentacosene

**Table 4:** Main compounds identified in *Hibiscus syriacus* leaf hydromethanolic extract by GC–MS.

Peak	R_t_ (min)	Area (%)	Assignments
1	6.078	2.51	urea, (1,1-dimethylethyl)-
4	17.154	2.87	phytol, acetate
7	18.021	1.86	hexadecanoic acid, methyl ester
8	18.396	2.38	*n*-hexadecanoic acid
9	19.662	2.49	9,12-octadecadienoic acid, methyl ester
10	19.725	2.49	9,12,15-octadecatrienoic acid, methyl ester
11	19.842	9.03	phytol
13	23.055	1.13	pentacosane
14	23.177	3.09	hexadecanoic acid, 2-hydroxy-1-(hydroxymethyl)ethyl ester
15	24.555	3.85	heptacosane
16	24.618	1.42	9,12,15-octadecatrien-1-ol
17	25.538	2.96	squalene
18	25.957	3.87	octadecane
20	28.002	15.97	vitamin E
21	29.092	1.63	campesterol
22	29.448	1.66	stigmasterol
23	30.154	5.75	sitosterol
25	31.575	1.16	vitamin E
26	31.867	23.05	3,4-dihydro-4,4,6,8-tetramethyl-coumarin (or 4,4,6,8-tetramethyl-2-chromanone)
27	32.111	2.11	6-isopropenyl-4,8a-dimethyl-3,5,6,7,8,8a-hexahydro-2(1*H*)-naphthalenone
28	32.802	3.46	selina-6-en-4-ol

**Table 5:** Antibacterial activity of chitosan oligomers (COS), *H. syriacus* flower and leaf hydromethanolic extracts, their main constituents (heptacosanol, DHTMC, and vitamin E), and their corresponding conjugate complexes (COS–flower extract, COS–leaf extract, COS–heptacosanol, COS–DHTMC, and COS–vitamin E) against the two phytopathogenic bacteria under study at different concentrations (expressed in μg·mL^−1^).

Pathogen	Compound	Concentration (μg·mL^−1^)									
		62.5	93.75	125	187.5	250	375	500	750	1000	1500
*E. amylovora*	COS	+	+	+	+	+	+	+	+	+	−
	Flower extract	+	+	+	+	+	+	+	−	−	−
	Leaf extract	+	+	+	+	+	+	+	+	−	−
	Heptacosanol	+	+	+	+	+	+	+	+	+	−
	DHTMC	+	+	+	+	+	+	+	+	−	−
	Vitamin E	+	+	+	+	+	+	+	−	−	−
	COS–Flower extract	+	+	+	+	+	+	−	−	−	−
	COS–Leaf extract	+	+	+	+	+	−	−	−	−	−
	COS–Heptacosanol	+	+	+	+	+	+	+	+	−	−
	COS–DHTMC	+	+	+	+	+	+	−	−	−	−
	COS–Vitamin E	+	+	+	+	-	-	-	-	-	-
*E. vitivora*	COS	+	+	+	+	+	+	+	+	+	−
	Flower extract	+	+	+	+	+	+	−	−	−	−
	Leaf extract	+	+	+	+	+	+	+	+	+	−
	Heptacosanol	+	+	+	+	+	+	−	−	−	−
	DHTMC	+	+	+	+	+	+	+	−	−	−
	Vitamin E	+	+	+	+	+	+	−	−	−	−
	COS–Flower extract	+	+	+	+	−	−	−	−	−	−
	COS–Leaf extract	+	+	+	+	+	+	−	−	−	−
	COS–Heptacosanol	+	+	+	−	−	−	−	−	−	−
	COS–DHTMC	+	+	+	−	−	−	−	−	−	−
	COS–Vitamin E	+	+	+	+	-	-	-	-	-	-

DHTMC = 3,4-dihydro-4,4,6,8-tetramethyl-coumarin.

**Table 6:** EC_50_ and EC_90_ effective concentrations of *H. syriacus* flower and leaf extracts and their phytochemicals against *D. seriata*, alone and upon conjugation with chitosan oligomers (COS).

EC	COS	Flower Extract	COS–Flower Extract	Leaf Extract	COS–Leaf Extract	Hepta	COS–Hepta	DHTMC	COS–DHTMC	Vit. E	COS–Vit. E
EC_50_	744.4	834.7	753.1	1053.3	301.0	187.6	122.4	452.1	217.7	237.3	217.4
EC_90_	1179.9	1530.5	975.8	2376.0	603.5	378.3	221.0	608.3	484.9	479.4	406.1

Hepta = heptacosanol; DHTMC = 3,4-dihydro-4,4,6,8-tetramethyl-coumarin.

**Table 7:** Synergy factors, estimated according to Wadley’s method, for the conjugate complexes under study.

EC	COS–Flower Extract	COS–Leaf Extract	COS–Heptacosanol	COS–DHTMC	COS–Vitamin E
EC_50_	1.07	2.90	2.45	2.58	0.89
EC_90_	1.37	2.61	2.59	1.66	3.14

DHTMC = 3,4-dihydro-4,4,6,8-tetramethyl-coumarin.

## Data Availability

The data presented in this study are available on request from the corresponding author. The data are not publicly available due to their relevance to be part of an ongoing PhD Thesis.

## Supplementary Materials

The following are available online at https://www.mdpi.com/article/10.3390/plants10091876/s1, Figure S1: DSC and TG curves of *Hibiscus syriacus* flowers. Figure S2: DSC and TG curves of *Hibiscus syriacus* leaves. Figure S3: GC–MS chromatogram of *Hibiscus syriacus* flower hydromethanolic extract. Figure S4: GC–MS chromatogram of *Hibiscus syriacus* leaf hydromethanolic extract. Figure S5: Growth inhibition of *D. seriata* for the conjugate complexes under study.

## References

[1] Vasudeva N., Sharma S. (2008). Biologically active compounds from the genus *Hibiscus*. Pharm. Biol..

[2] Maganha E.G., Halmenschlager R.D.C., Rosa R.M., Henriques J.A.P., Ramos A.L.L.D.P., Saffi J. (2010). Pharmacological evidences for the extracts and secondary metabolites from plants of the genus *Hibiscus*. Food Chem..

[3] Inikpi E., Lawal O.A., Ogunmoye A., Ogunwande I.A. (2014). Volatile composition of the floral essential oil of *Hibiscus sabdariffa* L. from Nigeria. Am. J. Essent. Oils Nat. Prod..

[4] Melecchi M.I.S., Martinez M.M., Abad F.C., Zini P.P., do Nascimento Filho I., Caramão E.B. (2002). Chemical composition of *Hibiscus tiliaceus* L. flowers: A study of extraction methods. J. Sep. Sci..

[5] Dingjian C., Dare Y., Qingxiu J., Hongming Z., Hui L. (2009). GC/MS analysis on composition of the essential oil from *Hibiscus syriacus* L.. Chin. Agric. Sci. Bull..

[6] Nandagopalan V., Gritto M.J., Doss A. (2015). GC-MS analysis of bioactive components of the methanol extract of *Hibiscus tiliaceus* Linn. Asian J. Plant Sci. Res..

[7] Rassem H., Nour A.H., Yunus R.M. (2017). GC-MS analysis of bioactive constituents of *Hibiscus* flower. Aust. J. Basic Appl. Sci..

[8] Jang Y.-W., Jung J.-Y., Lee I.-K., Kang S.-Y., Yun B.-S. (2012). Nonanoic acid, an antifungal compound from *Hibiscus syriacus* Ggoma. Mycobiology.

[9] Rao K., Geetha K., Banji D. (2014). Quality control study and standardization of *Hibiscus rosa-sinensis* L. flowers and leaves as per WHO guidelines. J. Pharmacogn. Phytochem..

[10] Subhaswaraj P., Sowmya M., Bhavana V., Dyavaiah M., Siddhardha B. (2017). Determination of antioxidant activity of *Hibiscus sabdariffa* and *Croton caudatus* in *Saccharomyces cerevisiae* model system. J. Food Sci. Technol..

[11] Kobaisy M., Tellez M.R., Webber C.L., Dayan F.E., Schrader K.K., Wedge D.E. (2001). Phytotoxic and fungitoxic activities of the essential oil of kenaf (*Hibiscus cannabinus* L.) leaves and its composition. J. Agric. Food Chem..

[12] Olivia N.U., Goodness U.C., Obinna O.M. (2021). Phytochemical profiling and GC-MS analysis of aqueous methanol fraction of *Hibiscus asper* leaves. Future J. Pharm. Sci..

[13] Mahadevan N., Kamboj P. (2009). Hibiscus sabdariffa Linn.—An overview. Nat. Prod. Radiance.

[14] Ishikura N. (1973). Anthocyanins and flavonols in the flowers of *Hibiscus mutabilis* F. versicolor. Kumamoto J. Sci. Biol..

[15] Zhang E., Kang Q., Zhang Z. (1993). Chemical constituents from the bark of *Hibiscus syriacus* L.. China J. Chin. Mater. Med..

[16] Kim J.H., Nonaka G.-I., Fujieda K., Uemoto S. (1989). Anthocyanidin malonylglucosides in flowers of *Hibiscus syriacus*. Phytochemistry.

[17] Wei Q., Ji X., Xu F., Li Q., Yin H. (2015). Chemical constituents from leaves of *Hibiscus syriacus* and their α-glucosidase inhibitory activities. J. Chin. Med. Mater..

[18] Zhang R.-R., Hu R.-D., Lu X.-Y., Ding X.-Y., Huang G.-Y., Duan L.-X., Zhang S.-J. (2020). Polyphenols from the flower of *Hibiscus syriacus* Linn ameliorate neuroinflammation in LPS-treated SH-SY5Y cell. Biomed. Pharmacother..

[19] Yun B.-S., Ryoo I.-J., Lee I.-K., Yoo I.-D. (1998). Hibispeptin B, a novel cyclic peptide from Hibiscus syriacus. Tetrahedron.

[20] Yun B.-S., Ryoo I.-J., Lee I.-K., Park K.-H., Choung D.-H., Han K.-H., Yoo I.-D. (1999). Two bioactive pentacyclic triterpene esters from the root bark of *Hibiscus syriacus*. J. Nat. Prod..

[21] Yoo I.-D., Yun B.-S., Lee I.-K., Ryoo I.-J., Choung D.-H., Han K.-H. (1998). Three naphthalenes from root bark of *Hibiscus syriacus*. Phytochemistry.

[22] Shi L.-S., Wu C.-H., Yang T.-C., Yao C.-W., Lin H.-C., Chang W.-L. (2014). Cytotoxic effect of triterpenoids from the root bark of *Hibiscus syriacus*. Fitoterapia.

[23] Punasiya R., Joshi A., Sainkediya K., Tirole S., Joshi P., Das A., Yadav R. (2011). Evaluation of antibacterial activity of various extracts of *Hibiscus syriacus*. Res. J. Pharm. Technol..

[24] Shah M., Bokadia M., Mehta B., Jain S. (1988). Chemical composition and antimicrobial activity of some seed oils. Fitoterapia.

[25] Yokota M., Zenda H., Kosuge T., Yamamoto T. (1978). Studies on isolation of naturally occurring biologically active principles. IV. Antifungal constituents in the bark of *Hibiscus syriacus* L.. J. Pharm. Soc. Jpn..

[26] Zhao Y.-Q., Tian Y.-L., Wang L.-M., Geng G.-M., Zhao W.-J., Hu B.-S., Zhao Y.-F. (2019). Fire blight disease, a fast-approaching threat to apple and pear production in China. J. Integr. Agric..

[27] Szegedi E., Civerolo E.L. (2011). Bacterial diseases of grapevine. Int. J. Hortic. Sci..

[28] Larignon P., Fulchic R., Cere L., Dubos B. (2001). Observation on black dead arm in French vineyards. Phytopathol. Mediterr..

[29] Mondello V., Songy A., Battiston E., Pinto C., Coppin C., Trotel-Aziz P., Clement C., Mugnai L., Fontaine F. (2018). Grapevine trunk diseases: A review of fifteen years of trials for their control with chemicals and biocontrol agents. Plant Dis..

[30] Stevens N.E. (2018). Two apple black rot fungi in the United States. Mycologia.

[31] Brown-Rytlewski D.E., McManus P.S. (2000). Virulence of *Botryosphaeria dothidea* and *Botryosphaeria obtusa* on apple and management of stem cankers with fungicides. Plant Dis..

[32] Brown E.A. (1986). *Botryosphaeria* diseases of apple and peach in the Southeastern Unites States. Plant Dis..

[33] Venditti A. (2018). What is and what should never be: Artifacts, improbable phytochemicals, contaminants and natural products. Nat. Prod. Res..

[34] Subramanian S., Reddy Ragula U.B. (2018). Pyrolysis kinetics of *Hibiscus rosa sinensis* and *Nerium oleander*. Biofuels.

[35] Ghetti P., Ricca L., Angelini L. (1996). Thermal analysis of biomass and corresponding pyrolysis products. Fuel.

[36] Meryemoğlu B., Hasanoğlu A., Irmak S., Erbatur O. (2014). Biofuel production by liquefaction of kenaf (*Hibiscus cannabinus* L.) biomass. Bioresour. Technol..

[37] Saba N., Jawaid M., Hakeem K.R., Paridah M.T., Khalina A., Alothman O.Y. (2015). Potential of bioenergy production from industrial kenaf (*Hibiscus cannabinus* L.) based on Malaysian perspective. Renew. Sustain. Energy Rev..

[38] Wong S., Lim Y., Chan E. (2010). Evaluation of antioxidant, anti-tyrosinase and antibacterial activities of selected *Hibiscus* species. Ethnobot. Leafl..

[39] Sim Y.Y., Jess Ong W.T., Nyam K.L. (2019). Effect of various solvents on the pulsed ultrasonic assisted extraction of phenolic compounds from *Hibiscus cannabinus* L. leaves. Ind. Crop. Prod..

[40] Čechovská L., Cejpek K., Konečný M., Velíšek J. (2011). On the role of 2,3-dihydro-3,5-dihydroxy-6-methyl-(4H)-pyran-4-one in antioxidant capacity of prunes. Eur. Food Res. Technol..

[41] Mak Y.W., Chuah L.O., Ahmad R., Bhat R. (2013). Antioxidant and antibacterial activities of hibiscus (*Hibiscus rosa-sinensis* L.) and Cassia (*Senna bicapsularis* L.) flower extracts. J. King Saud Univ. Sci..

[42] Seyyednejad S.M., Koochak H., Darabpour E., Motamedi H. (2010). A survey on *Hibiscus rosa—Sinensis*, *Alcea rosea* L. and *Malva neglecta* Wallr as antibacterial agents. Asian Pac. J. Trop. Med..

[43] Liu Q., Luyten W., Pellens K., Wang Y., Wang W., Thevissen K., Liang Q., Cammue B.P.A., Schoofs L., Luo G. (2012). Antifungal activity in plants from Chinese traditional and folk medicine. J. Ethnopharmacol..

[44] Imada C. (2005). Enzyme inhibitors and other bioactive compounds from marine actinomycetes. Antonie Van Leeuwenhoek.

[45] Vambe M., Aremu A.O., Chukwujekwu J.C., Gruz J., Luterová A., Finnie J.F., Van Staden J. (2020). Antibacterial, mutagenic properties and chemical characterisation of sugar Bush (*Protea caffra* Meisn.): A South African native shrub species. Plants.

[46] Zarger M.S.S., Akhtar N., Shreaz S., Bhatia R., Khatoon F. (2014). Phytochemical analysis and growth inhibiting effects of *Salix viminalis* L. Leaves against different *Candida* isolates. Adv. Sci. Lett..

[47] Pinto M.E.A., Araújo S.G., Morais M.I., Sá N.P., Lima C.M., Rosa C.A., Siqueira E.P., Johann S., Lima L.A.R.S. (2017). Antifungal and antioxidant activity of fatty acid methyl esters from vegetable oils. An. Acad. Bras. Ciências.

[48] Bhat M.Y., Talie M.D., Wani A.H., Lone B.A. (2020). Chemical composition and antifungal activity of essential oil of *Rhizopogon* species against fungal rot of apple. J. Appl. Biol. Sci..

[49] Vambe M., Naidoo D., Aremu A.O., Finnie J.F., Van Staden J. (2019). Bioassay-guided purification, GC-MS characterization and quantification of phyto-components in an antibacterial extract of *Searsia lancea* leaves. Nat. Prod. Res..

[50] Zheng C.J., Yoo J.-S., Lee T.-G., Cho H.-Y., Kim Y.-H., Kim W.-G. (2005). Fatty acid synthesis is a target for antibacterial activity of unsaturated fatty acids. FEBS Lett..

[51] Liu S., Ruan W., Li J., Xu H., Wang J., Gao Y., Wang J. (2008). Biological Control of Phytopathogenic Fungi by Fatty Acids. Mycopathologia.

[52] Mutlu-Ingok A., Devecioglu D., Dikmetas D.N., Karbancioglu-Guler F., Capanoglu E. (2020). Antibacterial, antifungal, antimycotoxigenic, and antioxidant activities of essential oils: An updated review. Molecules.

[53] De Souza S.M., Monache F.D., Smânia A. (2005). Antibacterial activity of coumarins. Z. Nat. C.

[54] Montagner C., de Souza S.M., Groposo C., Delle Monache F., Smânia E.F.A., Smânia A. (2008). Antifungal activity of coumarins. Z. Nat. C.

[55] Halbwirth H., Fischer T.C., Roemmelt S., Spinelli F., Schlangen K., Peterek S., Sabatini E., Messina C., Speakman J.-B., Andreotti C. (2003). Induction of antimicrobial 3-deoxyflavonoids in pome fruit trees controls fire blight. Z. Nat. C.

[56] Flachowsky H., Halbwirth H., Treutter D., Richter K., Hanke M.-V., Szankowski I., Gosch C., Stich K., Fischer T.C. (2012). Silencing of flavanone-3-hydroxylase in apple (*Malus× domestica* Borkh.) leads to accumulation of flavanones, but not to reduced fire blight susceptibility. Plant Physiol. Biochem..

[57] Vrancken K., Holtappels M., Schoofs H., Deckers T., Valcke R. (2013). Pathogenicity and infection strategies of the fire blight pathogen *Erwinia amylovora* in Rosaceae: State of the art. Microbiology.

[58] Du D., Wang Z., James N.R., Voss J.E., Klimont E., Ohene-Agyei T., Venter H., Chiu W., Luisi B.F. (2014). Structure of the AcrAB–TolC multidrug efflux pump. Nature.

[59] Al-Salih D.A.A.K., Aziz F.M., Mshimesh B.A.R., Jehad M.T. (2013). Antibacterial effects of vitamin E: In vitro study. J. Biotechnol. Res. Cent..

[60] Baran R., Thomas L. (2009). Combination of fluconazole and alpha-tocopherol in the treatment of yellow nail syndrome. J. Drugs Dermatol..

[61] Pejin B., Savic A., Sokovic M., Glamoclija J., Ciric A., Nikolic M., Radotic K., Mojovic M. (2014). Further in vitro evaluation of antiradical and antimicrobial activities of phytol. Nat. Prod. Res..

[62] Buzón-Durán L., Langa-Lomba N., González-García V., Casanova-Gascón J., Martín-Gil J., Pérez-Lebeña E., Martín-Ramos P. (2021). On the applicability of chitosan oligomers-amino acid conjugate complexes as eco-friendly fungicides against grapevine trunk pathogens. Agronomy.

[63] Langa-Lomba N., Buzón-Durán L., Martín-Ramos P., Casanova-Gascón J., Martín-Gil J., Sánchez-Hernández E., González-García V. (2021). Assessment of conjugate complexes of chitosan and *Urtica dioica* or *Equisetum arvense* extracts for the control of grapevine trunk pathogens. Agronomy.

[64] Santos-Moriano P., Fernandez-Arrojo L., Mengibar M., Belmonte-Reche E., Peñalver P., Acosta F.N., Ballesteros A.O., Morales J.C., Kidibule P., Fernandez-Lobato M. (2017). Enzymatic production of fully deacetylated chitooligosaccharides and their neuroprotective and anti-inflammatory properties. Biocatal. Biotransform..

[65] Buzón-Durán L., Martín-Gil J., Pérez-Lebeña E., Ruano-Rosa D., Revuelta J.L., Casanova-Gascón J., Ramos-Sánchez M.C., Martín-Ramos P. (2019). Antifungal agents based on chitosan oligomers, ε-polylysine and *Streptomyces* spp. secondary metabolites against three *Botryosphaeriaceae* species. Antibiotics.

[66] Van Laere K., Van Huylenbroeck J.M., Van Bockstaele E. (2007). Interspecific hybridisation between *Hibiscus syriacus*, *Hibiscus sinosyriacus* and *Hibiscus paramutabilis*. Euphytica.

[67] Winters H.F. (1970). Our hardy *Hibiscus* species as ornamentals. Econ. Bot..

[68] Punasiya R., Devre K., Pillai S. (2014). Pharmacognostic and pharmacological overview on *Hibiscus syriacus* L.. Int. J. Pharm. Life Sci..

[69] Martin M.T., Cobos R. (2007). Identification of fungi associated with grapevine decline in Castilla y León (Spain). Phytopathol. Mediterr..

[70] Talwalkar A.T. (1981). IGT/DOE Coal-Conversion Systems Technical Data Book.

[71] Dudonné S., Vitrac X., Coutière P., Woillez M., Mérillon J.M. (2009). Comparative study of antioxidant properties and total phenolic content of 30 plant extracts of industrial interest using DPPH, ABTS, FRAP, SOD, and ORAC assays. J. Agric. Food Chem..

[72] CLSI (2018). Methods for Dilution Antimicrobial Susceptibility Tests for Bacteria That Grow Aerobically.

[73] Arendrup M.C., Cuenca-Estrella M., Lass-Flörl C., Hope W. (2012). EUCAST technical note on the EUCAST definitive document EDef 7.2: Method for the determination of broth dilution minimum inhibitory concentrations of antifungal agents for yeasts EDef 7.2 (EU-CAST-AFST). Clin. Microbiol. Infect..

[74] Levy Y., Benderly M., Cohen Y., Gisi U., Bassand D. (1986). The joint action of fungicides in mixtures: Comparison of two methods for synergy calculation. EPPO Bull..

