# Supplementary material for: Physicochemical Characterization and Antimicrobial Activity against Erwinia amylovora, Erwinia vitivora, and Diplodia seriata of a Light Purple Hibiscus syriacus L. Cultivar
Source: Plants (Basel). 2021 Sep 10;10(9):1876. doi: 10.3390/plants10091876 (PMC8468437; doi:10.3390/plants10091876)
Supplement: Supplementary file 1 [file plants-10-01876-s001.zip › plants-1353160-supplementary.pdf]

Physicochemical characterization and antimicrobial activity against *Erwinia amylovora*, *Erwinia vitivora,* and *Diplodia seriata* of a light purple *Hibiscus syriacus* L. cultivar

E. Sánchez-Hernández, L. Buzón-Durán, B. Lorenzo-Vidal, J. Martín-Gil and P. Martín-Ramos

SUPPORTING INFORMATION

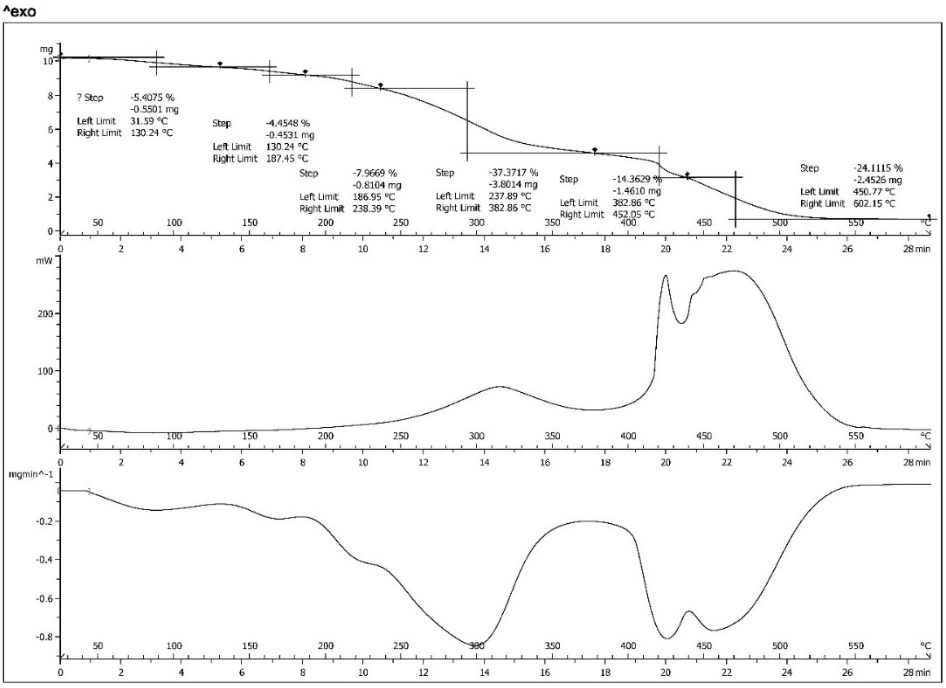

Figure S1. DSC and TG curves of *Hibiscus syriacus* flowers

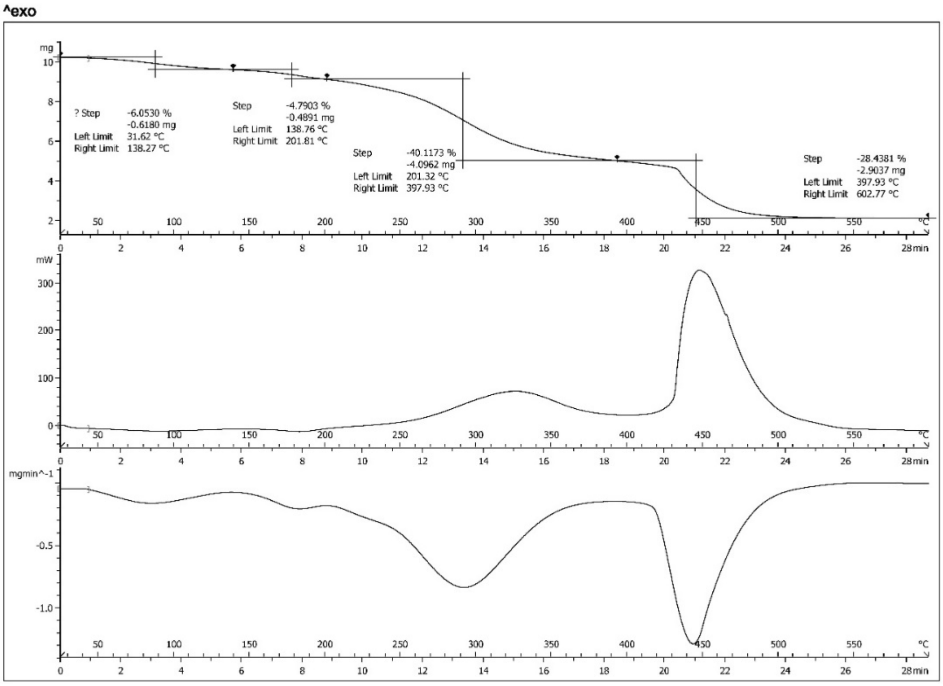

Figure S2. DSC and TG curves of *Hibiscus syriacus* leaves

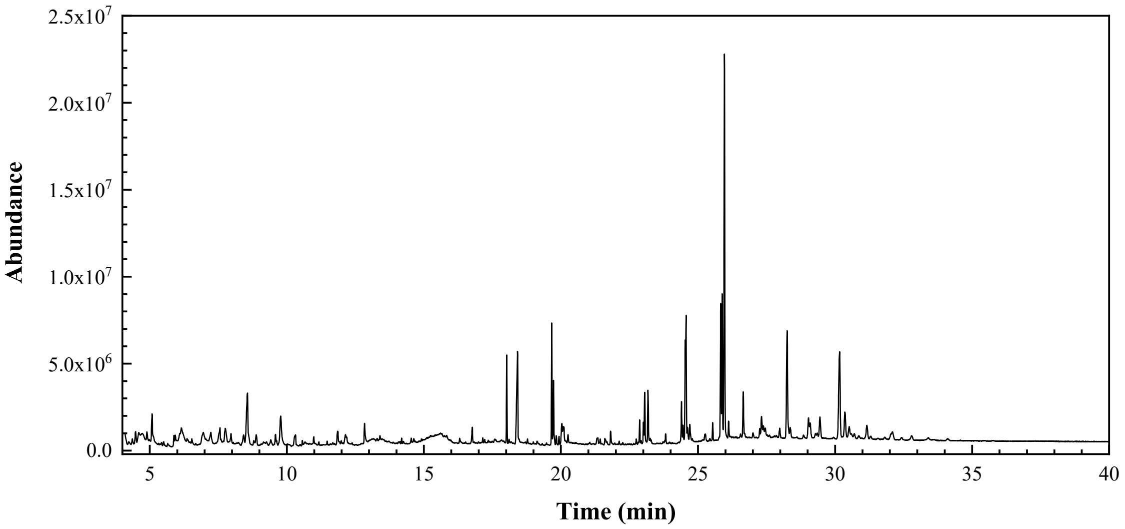

Figure S3. GC−MS chromatogram of *Hibiscus syriacus* flower hydromethanolic extract

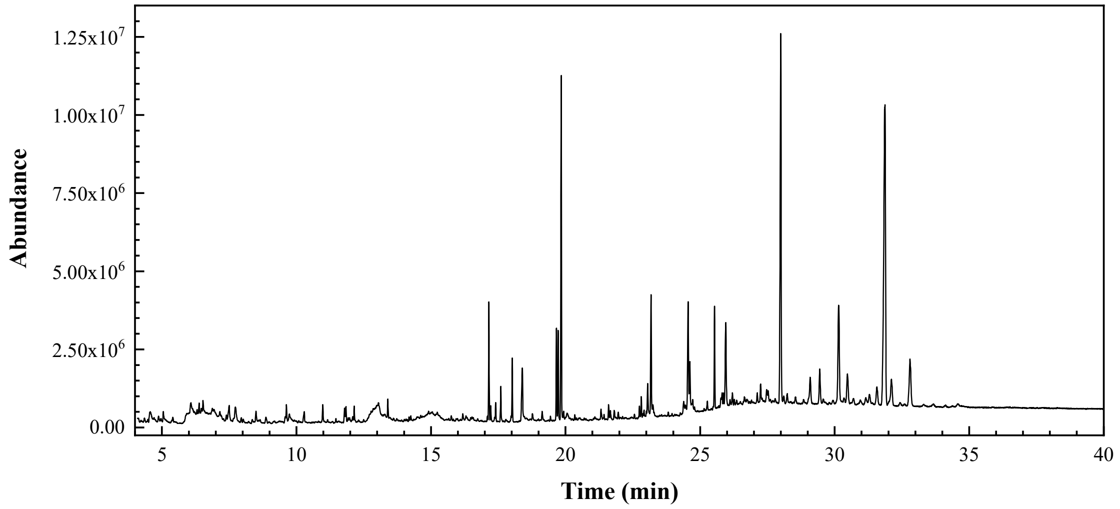

Figure S4. GC−MS chromatogram of *Hibiscus syriacus* leaf hydromethanolic extract

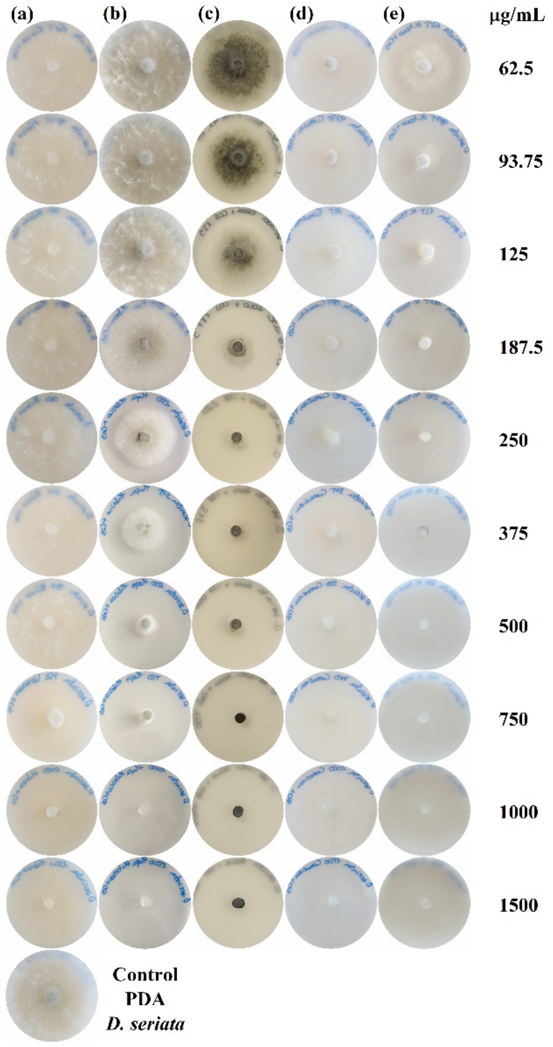

Figure S5. Growth inhibition of *D. seriata* for the conjugate complexes under study: **(a)** COS–flower extract; **(b)** COS–leaf extract; **(c)** COS–heptacosanol; **(d)** COS–DHTMC; **(e)** COS–vitamin E. Treatment doses from top to bottom: 62.5, 93.75, 125, 187.5, 250, 375, 500, 750, 1000 and 1500 $\mu g \cdot mL^{-1}$. The control (PDA medium without amendments) is shown in the bottom left corner. Only one replicate is shown.
